# Tissue specific characteristics of cells isolated from human and rat tendons and ligaments

**DOI:** 10.1186/1749-799X-3-32

**Published:** 2008-07-24

**Authors:** N Scutt, CG Rolf, A Scutt

**Affiliations:** 1 Dept. of Engineering Materials, Sir Robert Hadfield Building, Mappin Street, Sheffield, S1 3JD, UK; 2 Sheffield Centre of Sports Medicine, 5 Broomfield Road, Sheffield, S10 2SE, UK; 3 Section of Musculoskeletal Science, School of Medicine and Biomedical Sciences, University of Sheffield, Beech Hill Road, Sheffield, S10 2RX, UK

## Abstract

**Background:**

Tendon and ligament injuries are common and costly in terms of surgery and rehabilitation. This might be improved by using tissue engineered constructs to accelerate the repair process; a method used successfully for skin wound healing and cartilage repair. Progress in this field has however been limited; possibly due to an over-simplistic choice of donor cell. For tissue engineering purposes it is often assumed that all tendon and ligament cells are similar despite their differing roles and biomechanics. To clarify this, we have characterised cells from various tendons and ligaments of human and rat origin in terms of proliferation, response to dexamethasone and cell surface marker expression.

**Methods:**

Cells isolated from tendons by collagenase digestion were plated out in DMEM containing 10% fetal calf serum, penicillin/streptomycin and ultraglutamine. Cell number and collagen accumulation were by determined methylene blue and Sirius red staining respectively. Expression of cell surface markers was established by flow cytometry.

**Results:**

In the CFU-f assay, human PT-derived cells produced more and bigger colonies suggesting the presence of more progenitor cells with a higher proliferative capacity. Dexamethasone had no effect on colony number in ACL or PT cells but 10 nM dexamethasone increased colony size in ACL cultures whereas higher concentrations decreased colony size in both ACL and PT cultures. In secondary subcultures, dexamethasone had no significant effect on PT cultures whereas a stimulation was seen at low concentrations in the ACL cultures and an inhibition at higher concentrations. Collagen accumulation was inhibited with increasing doses in both ACL and PT cultures. This differential response was also seen in rat-derived cells with similar differences being seen between Achilles, Patellar and tail tendon cells. Cell surface marker expression was also source dependent; CD90 was expressed at higher levels by PT cells and in both humans and rats whereas D7fib was expressed at lower levels by PT cells in humans.

**Conclusion:**

These data show that tendon & ligament cells from different sources possess intrinsic differences in terms of their growth, dexamethasone responsiveness and cell surface marker expression. This suggests that for tissue engineering purposes the cell source must be carefully considered to maximise their efficacy.

## Background

Tendon and ligament injuries are very common in sports as well as in sedentary population, comprising chronic pain conditions, and acute complete or partial ruptures [1]. Treatment of tendon and ligament injuries is costly in terms of both the time taken for the repair process and the cost of surgery and rehabilitation. It has been estimated that about 30,000 tendon repair processes take place annually in the USA costing billions of dollars in their evaluation and management [2]. One possible way to improve this situation is to generate cell-seeded tissue engineered constructs to speed the repair process [3]. This line of research has proved successful in the fields of skin wound healing and cartilage repair [4,5]. However, although the relatively simple structure of tendons and ligaments and their minimal vascularisation would suggest that tendons and ligaments are ideal candidates for this type of treatment, progress in this field has been limited and has not progressed to the clinic. One reason for this might be an over-simplistic choice of cell type for incorporation into the tissue engineered constructs. To date a variety of cell types have been used including MSCs, dermal fibroblasts and cells extracted from tendons and ligaments themselves, with varying amounts of success [6-8]. To date the vast majority of tissue engineered tendons have been populated using MSCs and although a small number of studies have been published using tenocytes populated constructs, to our knowledge no attention has been paid to the exact source of the tenocytes.

Although it is often assumed that tendon and ligament-derived cells are similar, regardless of source, this is by no means the case and it may well be that the choice of cell type is an important factor in the successful generation of different tendon/ligament tissue engineered constructs. Differing matrix properties between tendons have been well described. For example Rumian et al [9] showed differences in collagen fibril diameter distribution, water content, GAG content and collagen dry weight between ligaments and tendons of the ovine hind limb. It is well documented that there are functional differences between tendons depending on their role in the body. Tendons can be classified according to their function into positional, which are relatively fixed, and energy storing which are involved in activities such as running, and are therefore subjected to much higher strains during activity, and these differences are reflected in differences in the matrix of the tendons [10]. Matrix turnover (degradation of damaged collagen by MMPs, and its replacement by newly synthesised collagen) in turn is a function of the cells present in the tendon. Whether phenotypic variation between tendon cells leading to alterations in matrix production occurs as a result of stimuli in the individual tendon microenvironment or is intrinsic to the individual tendon is unclear.

Although the literature is limited, tendon cells have been shown to express a number of markers including the human fibroblastic markers D7-FIB [11,12] and CD90 (data not shown) as well as other markers characteristic of mesenchymal cells (CD13 & CD44) (data not shown). The degree of expression of these markers by tendon and ligament cells has not been thoroughly investigated. However, differences in the intensity of expression of CD90 have been seen in other mesenchymal tissues; e.g. between human bone marrow or synovial-derived MSCs with the expression being higher in cells derived from the synovium [13]. The expression of this marker has also been shown to lose intensity in cultures of human MSC subjected to mechanical strain [14]. As MSC and tendon/ligament cells are phenotypically closely related, it would seem likely that a similar tissue/context specific expression will occur in tendons.

The response to treatment with glucocorticoids is another area where tendon/ligament cells appear to show some degree of site specificity. Glucocorticoids are used to treat a number of overuse-induced tendinoses however, the response to this form of treatment is variable and may result in recovery or induce side effects leading to rupture [15-18]. Clinical trials investigating the effectiveness of glucocorticoid therapy show varying results depending on the tendon treated. [19-22]. This diverse range of responses to glucocorticoid therapy may be due to a number of factors including type/preparation of drug used, its dosage and also the sensitivity of the various tendons and resident cell populations to glucocorticoids.

In vitro studies on the effects of corticosteroids on cultured tendon/ligament cells are also bedevilled by varying conditions of dosage, time of culture and usage of different tendons and ligaments from various species making direct comparisons difficult. Wong et al found a dexamethasone-induced reduction in collagen synthesis, cell proliferation and proteoglycan synthesis in passaged human patellar tenocytes [23,24]. In contrast Fermor et al found a dexamethasone-induced increase in cell proliferation and collagen synthesis in ACL-derived cells [25]. In vitro studies on rat tendons have all shown reduction in cell proliferation in the presence of dexamethasone but again direct comparisons between studies are difficult due to varying doses and culture conditions [26-28]. We have previously shown that in long term culture of rat tail tendon cells in the presence of dexamethasone, there is a concentration dependant decrease in cell number and collagen accumulation as compared to control cultures. We also showed that increasing doses of dexamethasone lead to decreasing colony size and above dexamethasone concentrations of 10 nM there was also a decrease in colony number in the fibroblastic -colony forming unit assay indicating reduction in progenitor cell recruitment in the presence of increasing dexamethasone concentrations [29]. However, the relevance of these data to human tendons remains unclear.

As the majority of in vitro studies have used a single tendon or ligament, tested in isolation, it is not possible to draw conclusions regarding the relative behaviour of different tendons and ligaments. This is particularly critical in relation to the use of tenocytes in a cell therapy or tissue engineering context. It is likely that not all tendons or ligaments will be prove to be optimal cell sources for therapeutic purposes. We therefore decided to compare cells derived from a range of human and rat tendons and ligaments in terms of their progenitor cell population, responsiveness to dexamethasone, and their expression of cell surface markers.

## Methods

### Reagents and consumables

Unless stated otherwise, all chemicals were purchased from Sigma-Aldrich (Poole, Dorset U.K.), tissue culture media from Lonza (Wokingham, U.K.) and plasticware from Nunc (Nottingham, U.K.), or Greiner Bio One (Gloucester, U.K) and used as supplied.

### Tissue samples and preparation

Human patellar tendon (PT) and anterior cruciate ligament (ACL) samples were collected from The Sheffield Centre for Sports Medicine during operations for the repair of ACL rupture. Samples were donated following informed patient consent and with local Ethical Committee approval. The samples were all from young male donors with an age range of 18–23 (mean age 20). The choice of males with as tight an age range as possible was to avoid possible age or gender related effects, both of which are known to affect tendon pathophysiology [30-33]. Samples were collected into tissue culture medium (DMEM) without additives and then transported directly to the laboratory.

Rat tendon samples were collected from male Wistar rats, (200–250 g) that were killed by a schedule 1 method. The PT from each knee was dissected free from the surrounding tissues with sharp scissors and both tendons combined for digestion. The Achilles tendons were cut free at the Calcanius and the adjoining muscles with scissors and combined for digestion. The tails were removed and the tendon fascicles were dissected from the surrounding tissues and combined. Under sterile conditions tissue samples were rinsed in DMEM, and then diced into small pieces and digested in sterile crude collagenase solution (Sigma crude collagenase from Clostridium histolyticum 1 mg/ml). The samples were incubated for 18 h at 37°C on a rotary blood mixer, after which time the majority of the collagen in the sample was digested and the cells freed into the medium. Following digestion, the sample was filtered through a 70 micron sieve, washed and assessed for cell number and vitality using the Guava Viacount system (Guava Technologies, Stamford, UK). After digestion, the tendon-derived cells (TDC) typically had a viability of greater than 90%.

### Fibroblastic-colony forming unit cultures

Fibroblastic-colony forming unit cultures (CFU-f) were performed as described previously for the investigation of CFU-f in bone marrow cells but with modifications [29]. Briefly, 1 × 10^3 ^primary TDC were plated out in 55 cm^2 ^petri dishes in Dulbecco's modified Eagle's medium containing 10% fetal calf serum pen/strep, ultraglutamine and 50 μg/ml ascorbate-2-phosphate and an appropriate concentration of dexamethasone. The medium was replaced after 5 days and thereafter twice weekly. Fresh supplements were added each time the culture medium was replenished. The cultures were maintained for 11 days after which time the cells were washed with PBS and fixed by the addition of cold ethanol. After fixation, the cultures were stained for total colonies with 0.1% methylene blue in 10 mM borate buffer for 30 min, excess stain was then removed by washing under running tap water. The cultures were dried and photographed, then analyzed using "Gene Tools" image analysis software (Syngene, Cambridge, UK) and the number and size of colonies calculated.

### High density cultures

Tendon or ligament-derived cells were expanded in tissue culture flasks in DMEM plus 10% fetal calf serum pen/strep, and ultraglutamine for up to 2 passages. They were then plated into 24 well plates at a density of 5 × 10^3 ^cells per well in 0.5 ml medium containing ascorbate-2-phosphate and varying doses of dexamethasone. The medium was changed twice weekly. Fresh dexamethasone was added at each media changed. After one or two weeks, the cultures were washed with PBS and fixed using ethanol and analysed as described below.

### Collagen accumulation

Total collagen was assessed using a modification of the method of Lopez-de Leon and Rojkind [34]. After fixation, the cell layers were stained with 0.1% Sirius red F3BA in saturated picric acid for 18 h, after which excess Sirius red was removed by washing under running tap water. In high-density cultures, the dye was then eluted with 0.1 N NaOH/methanol (50:50), and the collagen quantitated by measuring spectrophotometrically at 490 nm.

### Cell number

Cell number was assessed by the method of Currie [35]. After fixation, the cells were washed with borate buffer (10 mM, pH 8.8), stained with 0.1% methylene blue in borate buffer for 30 min, and then rewashed three times with borate buffer. Bound methylene blue was eluted with 1% HCl in ethanol, and the absorbance measured at 650 nm.

### Flow cytometric analysis of cell surface markers

Flow cytometry was used to assess the expression of cell surface markers on tendon-derived cells. Antibodies were used at the manufacturers recommended dilutions and were purchased from Serotec (Oxford). Cells were used after one passage and were resuspended in microcentrifuge tubes at a density of 1 × 10^4 ^cells in 20 μl of medium. 20 μl of the appropriate primary antibody was added to the vial and the cells were then incubated at 4°C for 30 minutes. At the end of this time the cells were washed and incubated with a PE conjugated secondary antibody at 4°C for a further 20 minutes. The cells were again washed then analysed in the Guava Personal Cytometry system using the 'Protein Express' software package, analysing at least 5000 cells per sample.

### Data handling and statistical analyses

Data are presented as group means +/- standard deviations. At least three replicates of each experiment were performed, and the results presented in the figures are representative of these. For each variable, effects across treatment groups were compared by one-way analysis of variance (ANOVA) using SPSS14.0 software. If the overall difference was significant, multiple comparisons were performed between groups using Tukey's test. Differences are considered significant at a probability of <0.05 on a two tailed test.

## Results

In order to compare human tendon/ligaments from different sources, cells from pairs of ACL and PTs isolated from the same subjects and during the same procedure were isolated. They were then compared in terms of their proliferation, collagen accumulation, levels of progenitor cells and response to dexamethasone.

**Figure 1:**
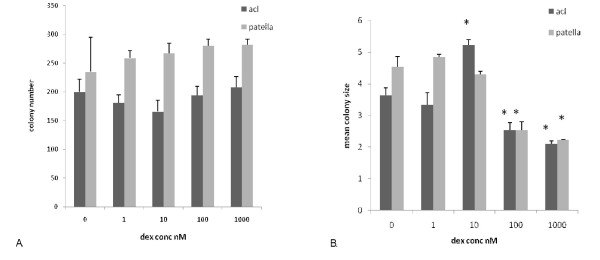
**Effect of dexamethasone on colony formation by primary human patellar or anterior cruciate ligament derived cell.** 1 × 10^3 ^cells were cultured in 56 cm^2 ^Petri dishes for 11 days as described in the text. The cultures were then stopped, stained with methylene blue and colony number (A) and colony size (B) determined by image analysis. * denotes statistical significance compared to appropriate control cultures, p < 0.05.

Cells from 3 pairs of PT and ACLs were examined for their levels of progenitor cells and their response to a range of concentrations of dexamethasone using the CFU-f assay. A typical example is shown in fig 1. The data shows that dexamethasone had no significant effect on the number of colonies formed over a range of concentrations in either the ACL samples or the PT samples. There were however, significantly more colonies per 1000 cells seeded in the patellar sample than in the ACL sample, indicating a higher proportion of proliferative cells in this tissue (Fig. 1A) and in addition, the patellar samples produced bigger colonies in the control and 1 nM cultures indicating a higher proliferative capacity (Fig. 1B). Although these results indicate that dexamethasone did not affect progenitor cell recruitment in terms of colony number, there was a differential effect on subsequent colony expansion. When the mean colony size was calculated, it was found that in the patellar samples there was a significant reduction in mean colony size at 100 and 1000 nM dexamethasone indicating a reduction in cell proliferation at these concentrations. Similarly, dexamethasone caused a reduction in colony size in the ACL samples at 100 and 1000 nM as compared with the controls, however, at 10 nM dexamethasone lead to a significant increase in mean colony size (Fig. 1B). Although this concentration response relationship was somewhat unexpected, it was reproducible and in 5 ACL samples, the mean colony size at 10 nM dexamethasone ranged from between 111% and 209% (mean 155%) of the mean control colony size (data not shown). Together these data suggest that dexamethasone at 10 nM (i.e. physiological levels) stimulated ACL cell proliferation but not that of the PT cells whereas at higher concentrations it repressed cell proliferation in both ACL and PT cells.

**Figure 2:**
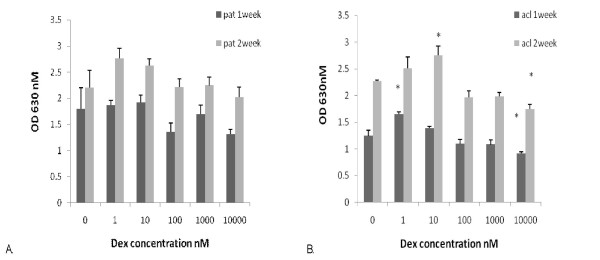
**Effect of dexamethasone on secondary human patellar (A) or anterior cruciate ligament (B) derived cell proliferation.** Cells were plated out in 24 well plates at a density of 5 × 10^3 ^cells per well as described above. The cultures were stopped after 1 or 2 weeks and cell number determined by methylene blue staining. * denotes statistical significance compared to appropriate control cultures, p < 0.05.

In order to see if this response persisted in culture, we looked at the response of secondary human tendon/ligament cells to dexamethasone using high density cultures in a series of 3 paired samples. 'The cells were used after 2 passages and cell number and collagen accumulation were measured after 1 and 2 weeks in culture; the results of a typical experiment are shown in fig 2. In the sub-cultured PT cells, cell proliferation, as measured by methylene blue staining, after one week in culture showed no significant changes although there was a slight downward trend at the higher doses tested (Fig. 2A). However after 2 weeks of culture, cells in the presence of dexamethasone at 1 nM showed a slight increase in proliferation as compared to the control cultures but this increase was not significant at the higher doses of dexamethasone (Fig. 2A). These results differ from those obtained in the primary CFU-f cultures where the higher concentrations of dexamethasone lead to a reduction in cell proliferation as measured by mean colony size (Fig. 1B). This may be a result of different culture conditions or a difference between primary and cultured PT cells.

In the passaged ACL cultures, again there was a significant increase in cell number in the presence of low doses dexamethasone as compared to control cultures. After 1 week of culture this was greatest at 1 nM dexamethasone but after 2 weeks of culture the peak was at 10 nM dexamethasone (Fig. 2B). However at both time points, the increase in cell number was not as large as that found in the ex vivo CFU-f cultures, again suggesting different sensitivities to dexamethasone using the different culture methods.

**Figure 3:**
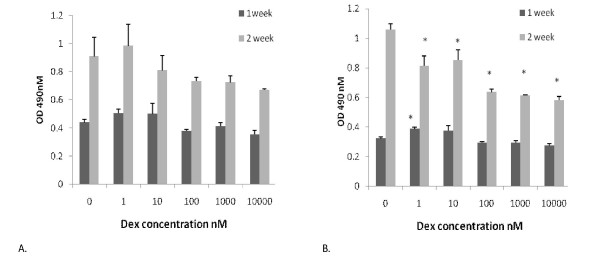
**Effect of dexamethasone on secondary human patellar (A) or anterior cruciate ligament (B) derived collagen accumaulation.** Cells were cultured as in Figure 2 and stopped after 1 or 2 weeks at which point collagen accumulation was determined by Sirius red staining. * denotes statistical significance compared to appropriate control cultures, p < 0.05.

Collagen accumulation in the PT cell cultures followed the same trend as the cell proliferation in that at both time points there was a slight but insignificant increase at low dexamethasone concentrations and a decrease at higher dexamethasone concentrations, probably reflecting the numbers of cells per culture. However the rate of increase between the first and second week of culture was far greater for collagen accumulation than for cell number with a 100% increase in the amount of collagen compared with about 25% increase in cell number in the control cultures suggesting a process of differentiation and specific increase in collagen accumulation (Fig. 3A).

In the ACL cultures, once again at week one the pattern of collagen accumulation paralleled changes in cell number indicating that the rate of collagen accumulation per cell number was constant over the range of dexamethasone doses. However, after 2 weeks in culture the pattern changed in that the control cultures showed a proportionally greater increase in collagen accumulation than the PT cultures. Furthermore, no stimulation was seen at low dexamethasone concentrations although, as in the 1 week cultures, an inhibition was seen at higher concentrations (Fig. 3B).

It was evident that cells derived from ACLs and PTs displayed a differential response to dexamethasone. However, as the PT samples used in these experiments were healthy (trimmings taken from tissue used for ACL autografts) whereas the ACL samples were derived from ruptured tissue, it was thought that these differences might reflect pathological changes rather than physiological differences between tendon/ligament types. To examine this possibility we then compared the responses to dexamethasone in 3 types of rat tendons derived from healthy rats.

**Figure 4:**
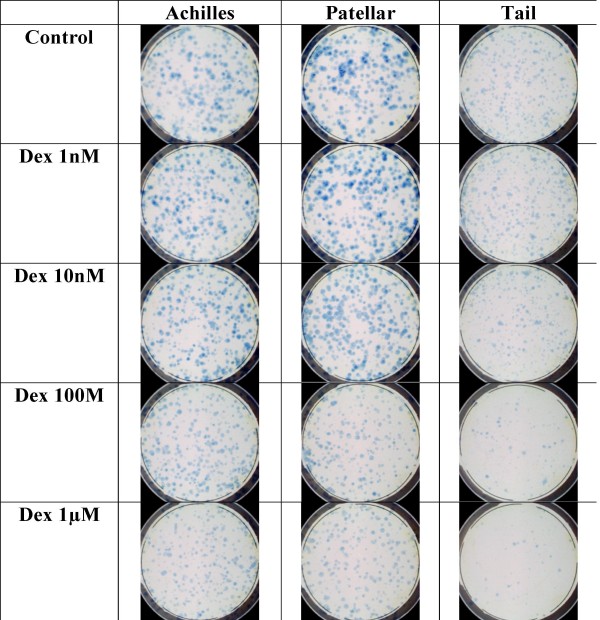
**Effect of dexamethasone on colony formation by primary rat Achilles, patellar or tail tendon derived cells.** 1 × 10^3 ^cells were cultured in 56 cm^2 ^Petri dishes for 11 days as described above and then stopped, stained with methylene blue and a digital image acquired.

**Figure 5:**
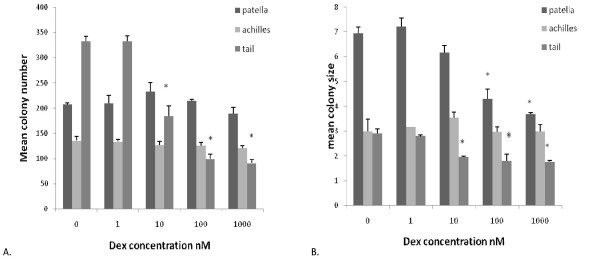
**Effect of dexamethasone on colony formation by primary rat Achilles, patellar or tail tendon-derived cell.** Cells were cultured as Figure 1 and then colony number (A) and colony size (B) determined by image analysis. * denotes statistical significance compared to appropriate control cultures, p < 0.05.

When cells derived from patellar, Achilles and tail tendons from 3 male Wistar rats were examined for their response to dexamethasone in the CFU-f assay, it was evident that the cells behaved differently in their growth and response to dexamethasone. Examples of the cultures are shown in Figure 4. On analysis, it was found that in terms of mean colony number, only the tail tendon cells showed any significant differences over the dexamethasone concentrations tested, with a significant reduction in colony number with doses of 100 nM or higher (Fig. 5A). The tail tendon results are in agreement with our previous findings [29] however, the PT cells showed no reduction in colony number over the range of doses tested, a similar result to the human patellar samples; and the Achilles tendon cells also showed no reduction in colony number (Fig. 5A). With regards to mean colony size it was evident that in the absence of dexamethasone the largest colonies (and therefore having the greatest rate of growth) was seen in the PT cells (Fig. 5B). In the tail cells, as before, there was a reduction in mean size at doses of 10 nM and above. In the PT cultures there was also a reduction in mean colony size, occurring at doses of 100 nM and above, again similar to the results found in the human samples. Interestingly, in the Achilles cells, although there was a reduction in the intensity of staining indicating a reduction in cell number, on analysis there was no significant reduction in colony size at any of the doses tested. These results, along with the human results suggest that the differences in sensitivity to dexamethasone are not due to the disease status of the tendons but rather due to specific differences in sensitivity in the various tendon tissues.

To see whether there were any intrinsic differences between cells derived from the different tendons, we determined the intensity of expression of cell surface markers typical of mesenchymal cells on cells cultured from all tendons used in the above experiments using flow cytometry. Rat cells were analysed for CD44 and CD90 and human cells for CD44, CD90 and D7fib. These markers were chosen as they are expressed by between 90 and 100% of cells from all sources facilitating accurate comparison of intensity data between cell types (Tables 1 and 2).

**Table 1:** Intensity of Expression of cell surface markers on rat patellar, Achilles or tail tendon-derived cells.

**Marker**	**PT**	**Achilles**	**Tail**
CD44	39.4 ± 4.3	42.8 ± 3.5	35.3 ± 3.9
CD90	105.9 ± 9.6	81.4 ± 5.1	83.2 ± 6.3

**Table 2:** Expression of cell surface markers on Human patellar tendon or anterior cruciate ligament cells.

	Patient 1		Patient 2		Patient 3		Patient 4	
	PT	ACL	PT	ACL	PT	ACL	PT	ACL

CD44	25.3	37.3	297.5	203.7	329.4	316.1	92.8	49.3
CD90	62.2	37.8	274.7	59.5	176.6	100.9	76.2	42
D7fib	42.2	74.1	42.6	105.9	50.5	72.2	43.1	49.8

Interestingly in both the rat and the human patellar samples, the marker CD90 showed a higher intensity of expression than in the ACL (human) or Achilles and tail (rat). In the rat there were no other obvious differences in the markers tested. In the human samples, the levels of expression varied greatly between patients, however, the general pattern of expression remained the same throughout. It was found that in all cases, ACL samples had a higher intensity of expression of the fibroblastic marker D7fib and a reduced CD90 expression compared to the patellar samples in all four pairs tested, however there were no consistent differences in CD13 or CD44.

## Discussion

In this manuscript we have demonstrated differences between tenocytes derived from tendons and ligaments from different sources in terms of their proliferation, collagen deposition, levels of progenitor cells, responses to dexamethasone and expression of cell surface markers. It is a sad fact that, despite considerable evidence to the contrary, tendons and ligaments tend to be considered to metabolically and cell biologically similar if not identical. In the clinic, treatment regimes often aim to treat only parts of the pathology, such as neovascularisation, that is similar for different structures without taking into account possible tissue specific differences [36]. Although histopathologic features are similar between different conditions, such as rotator cuff tears, patella tendinosis, Achilles tendinosis and "lateral epicondylitis" of the elbow, this does not necessarily mean that the response to treatment will be similar. Differences between tendons & ligaments and between flexor and extensor tendons have been referenced. However, this approach is probably too simplistic and given the different biomechanical stresses inflicted on tendons combined with the differences in vasculature and environment, it is likely that all tendons will be to some degree unique in terms of their cell biology and metabolism. Such differences are likely to have considerable relevance to therapies used to treat various tendinopathies, in particular in terms of the response to glucocorticoids treatment and to our knowledge; this is the first study investigating such tendon specific effects in human or rat tissues.

There is conflicting data regarding the effects of dexamethasone on tendon-derived cells in vitro. For example, Fermor et al showed that dexamethasone at 10 nM stimulated the proliferation of cells extracted from ACL's [25] whereas Wong et al showed that at concentrations between 1 nM and 100 uM dexamethasone inhibited the proliferation of cells from healthy PTs [23]. This is consistent with the in vitro data presented here in that the responses to this drug differed between the PTs and ACLs from the same individual, cultured under identical conditions. In the CFU-f assay there were more colonies per 1000 cells seeded in the patellar as compared to the ACL control samples although this difference was not significant, indicating the presence of a greater percentage of proliferative progenitor cells. The PT colonies were also larger in the control cultures indicating a greater basal proliferative rate in this tissue. In the PTs, a decrease in cell proliferation, as indicated by colony size was seen in ex vivo cultures in the CFU-f assay at doses greater than 10 nM although there was no effect on colony number. Similarly, in high density cultures of passaged cells at one week there was a small but non-significant decrease in cell number at higher doses, but after two weeks of culture there was an increase in cell number at doses of dexamethasone below 10 nM. This difference may well be a product of the differing length of exposure to the drug – 2 weeks as compared to 4 days, indeed at one week our cultures did not show increases in cell number at lower doses of dexamethasone.

PT and ACL cultures derived from the same individuals showed a different pattern of response to dexamethasone. In the ACL cultures, colony size (and hence cell proliferation) showed a marked increase at 10 nM – the same dose used by Fermor et al [25]. However this stimulation of cell proliferation was only seen at this dose and an inhibition was seen at doses of 100 nM and more. A similar pattern of response was seen in the high density cultures although here the differences were smaller. In contrast, no such stimulation was seen on the PT cultures and these data show that these two cell populations do indeed respond in a different manner despite being cultured under identical conditions and derived from the same individual. This could indicate an intrinsic difference in dexamethasone sensitivity between the two tissues. However, an alternative explanation could be that the ACL tissue was from a rupture and the PT was from clinically healthy tissue and a repair response may be the cause of these differing results. We therefore decided to compare the responses of healthy rat tendons to dexamethasone.

In the rat we compared the responses of the patellar, Achilles and tail tendons to dexamethasone. Ideally, we would have used ACLs as well; however, due to their small size we were unable to extract sufficient cells from this tissue for accurate experiments. In the CFU-f assay, again in the control cultures the PTs produced the largest colonies, followed by the Achilles with the tail cells producing the smallest colonies, indicating that the PT cells proliferate at the fastest rate of the tissues tested, a result similar to that found in the human tissue. These PT cells also responded to dexamethasone in a similar manner to the human PT cells suggesting that there is no difference in the response to this drug in the PTs in the two species tested. When the response to dexamethasone was tested in the three different rat tendons, there was a difference in response between the tendon cells as only the tail tendon cells showed a decrease in progenitor cell recruitment (i.e. a decrease in colony number) with increasing concentrations on dexamethasone. However there was a dose dependant decrease in colony size in the PT and tail cells but not the Achilles cells at the doses tested. These results therefore show that in the rat as in the human, there is a difference in sensitivity to dexamethasone between tendon cells isolated from different tendons but from the same subject.

To further characterise any intrinsic differences in cells from different tendons we looked at the intensity of expression on cell surface markers. Tendon cells have been shown to express the human fibroblastic markers D7-FIB [11,12] and CD90 as well as other markers expressed by MSCs (eg. CD13 & CD44) (data not shown). Here we found that in a group of four PT/ACL pairs the intensity of expression of CD90 was consistently lower in the ACL as compared to the patellar and the expression of D7 FIB was consistently higher, whereas the pattern of response was inconsistent in the other markers. In the rat, the intensity of expression of CD90 was also higher in the PT cells as compared to the Achilles and tail cells. Differences in intensity of expression of CD90 have been seen between MSC's derived from synovial tissue and bone marrow MSCs obtained from humans with the expression being higher in cells derived from the synovium [13]. This marker has also been shown to lose intensity of expression in cultures of human MSC subjected to mechanical strain as compared to unstrained cultures [14]. Therefore a possible explanation for this lower level of expression in the ruptured ACL tissue is that these cells are exposed to higher levels of mechanical strain in vivo prior to rupture.

The results presented here clearly show that cells taken from different tendon/ligaments have different responsiveness to dexamethasone, and degree of expression of some cell surface markers. Such source specific differences have been shown for other connective tissues including osteoblasts [37,38], chondrocytes [39-41] and mesenchymal stem cells [38] and the data presented above is consistent with this. In the rat, tail tendon cells appear to be most sensitive to dexamethasone followed by cells from the PT and the Achilles being least affected. The responses in the PT appear to be similar for both rat and human, both in expression of CD90 and reactivity to dexamethasone, indicating therefore that this pattern of response is not to be species specific. In the human ACL the effects of dexamethasone concentrations produce a different pattern of response to that of the PT in that there is a proliferative response at 10^-8 ^M but an inhibition of cell number at higher doses. At present we can only speculate as to the mechanism underlying these effects. This might lie at the level of the glucocorticoid receptor; although this is derived from a single gene, alternative splicing and posttranslational modification give rise to multiple receptor isoforms which together with site specific co-activators is thought to be responsible for glucocorticoid tissue specificity [42,43]. The non-linear dose-response to dexamethasone seen in the human ACL samples is quite different to the responses seen in all other tendons or ligaments tests, human or rodent. It is however, similar to the bell shaped response of MSCs to dexamethasone, peaking at 10 nM followed by an inhibitory effect [44]. As all of the ACL samples were from ACL ruptures undergoing surgery it is likely that this response was due to extrinsic cells invading during the healing process. In support of this, it is now widely accepted that MSC are associated with microvasculature and are probably pericytic in nature [45]. The structure and biomechanical environment of a tendon varies greatly both within and between tendons [46] and is also likely that the cellular make-up of a tendon or ligament reflects this. Both the patellar tendon and ACL are weight bearing tissues; the ACL is under a chronic stress that is dependent on the posture whereas the patellar tendon experiences a more pulsatile loading related to the activity of the Quadriceps femorus. This environment will play a role in selecting the population of cells present in the tendon although exactly how and how long this phenotype persists in culture is unknown. It might also be expected that cells from ACL and Patellar tendons might respond differently to mechanical loading in vitro and this is currently a focus of investigation in our laboratory. One study comparing human finger flexor and extensor tendons suggested that there is no difference [47] whereas a similar investigation in horse flexor and extensor tendons suggested an increased responsiveness in extensor tendons [30] although these studies might not necessarily be relevant to the human ACL/Patellar situation.

## Conclusion

In conclusion, these data show that tendon & ligament-derived cells from different sources possess intrinsic differences in terms of their growth, dexamethasone responsiveness and cell surface marker expression. This is not unexpected as different tendons perform radically different functions and their cell populations must adapt their behaviour to reflect this. The current rapid developments in tissue engineering, in particular of mesenchymal tissues, holds great promise for the treatment of chronic and debilitating diseases. However, the source of cells for the generation of tissue engineered constructs must be carefully considered to maximise their engraftment and efficacy.

## Competing interests

The authors declare that they have no competing interests.

## Authors' contributions

NS: planning of experiments, majority of experimental work, preparation of manuscript; CGR: sourcing of human tissue, preparation of manuscript; AS: planning of experiments, some experimental work, preparation of manuscript.
